# Physical inactivity during leisure and school time is associated with the presence of common mental disorders in adolescence

**DOI:** 10.11606/s1518-8787.2020054001888

**Published:** 2020-11-23

**Authors:** Vanessa Roriz Ferreira, Thiago Veiga Jardim, Thaís Inácio Rolim Póvoa, Ricardo Borges Viana, Ana Luiza Lima Sousa, Paulo César Veiga Jardim

**Affiliations:** I Universidade Federal de Goiás Faculdade de Medicina GoiâniaGO Brasil Universidade Federal de Goiás. Faculdade de Medicina. Liga de Hipertensão Arterial. Goiânia, GO, Brasil; II Brigham & Women's Hospital Division of Cardiovascular Medicine BostonMA USA Brigham & Women's Hospital. Division of Cardiovascular Medicine. Boston, MA, USA; III Harvard TH Chan School of Public Health Center for Health Decision Science Department of Health Policy and Management BostonMA USA Harvard TH Chan School of Public Health. Department of Health Policy and Management. Center for Health Decision Science, Boston, MA, USA; IV Universidade Estadual de Goiás Faculdade do Esporte ESEFFEGO Laboratório de Fisiologia do Exercício GoiâniaGO Brasil Universidade Estadual de Goiás. Laboratório de Fisiologia do Exercício da Faculdade do Esporte ESEFFEGO. Goiânia, GO, Brasil

**Keywords:** Adolescent, Sedentary Behavior, Mental Disorders, Mental Health, Physical Education and Training

## Abstract

**OBJECTIVE:**

To investigate the association of physical inactivity in leisure and school time with common mental disorders during adolescence.

**METHODS:**

The sample consisted of 73,399 adolescents (12–17 years old), participants in the *Estudo de Riscos Cardiovasculares em Adolescentes* (Erica — Study of Cardiovascular Risks in Adolescents). This cross-sectional, national and school-based study was conducted in 2013 and 2014 in Brazilian municipalities with more than 100,000 inhabitants. Leisure time physical activity was categorized according to weekly practice volume, and adolescents were classified as active (≥ 300 minutes/week), inactive (0 minute/week) and insufficiently active (1–299 minutes/week). Sports practice and participation in physical education classes at school were also analyzed. The presence of common mental disorders was assessed based on the general health questionnaire, with a cutoff point greater than or equal to 3. Odds ratios (OR) were estimated using multiple logistic regression.

**RESULTS:**

The chance of common mental disorders was 16% higher in the group that reported being inactive (0 minute/week) at leisure time (OR = 1.16; 95%CI 1.06 (1.27). The prevalence of common mental disorders was higher among young people who did not practice sports (37.2% *vs.* 25.9%; OR = 1.14; 95%CI 1.04–1.25) and did not participate in physical education classes at school (39.5% *vs.* 29.6%; OR = 1.25; 95%CI 1.15–1.36). Insufficient physical activity (1–299 minutes/week) did not increase the OR of common mental disorders. Practicing physical activity during leisure time, regardless of duration and weekly frequency, reduced the chances of common mental disorders in this population by 26%.

**CONCLUSIONS:**

Physical inactivity during leisure and school time is associated with the presence of common mental disorders in adolescence. The results suggest that sports practice, school physical education and physical activity during leisure time, even without reaching the current recommendation, are related to the mental health of young people.

## INTRODUCTION

Epidemiological data show that up to 20% of children and adolescents suffer from a disabling mental illness and that about 50% of mental disorders start in adolescence^1^. Worldwide, neuropsychiatric disorders are the main cause of years of life lost due to disability in the age group between 10 and 24 years^2^. In Brazil, data from the *Estudo de Riscos Cardiovasculares em Adolescentes* (Erica — Study of Cardiovascular Risks in Adolescents) reported a high prevalence (30.0%) of common mental disorders in the young population^3^.

Common mental disorders, also known as minor psychiatric disorders or psychological distress, are mental disorders without psychotic character not contemplated in the diagnostic criteria of categorical classification systems. They are characterized mainly by the presence of symptoms of depression and anxiety, as well as nonspecific somatic symptoms. These disorders, which may be early manifestations of more serious psychiatric diseases, harm the adolescent's school life and social relations^4^^,^^5^.

Recent evidence has related symptoms of depression and anxiety to physical inactivity^6^^,^^7^. A cross-sectional study with Australian adolescents showed that boys with low level of physical activity are more prone to depressive symptoms^6^. On the other hand, longitudinal research conducted in Germany showed that young women (12–26 years), when practicing physical activity for longer than usual, felt less depressed in the next morning^7^.

Being active during adolescence also increases the chance of staying active in adulthood, prevents cardiovascular diseases, improves body composition and increases muscle strength, in addition to immediate benefits, such as well-being and greater self-esteem^8^. Nevertheless, in Brazil, one in four adolescents does not practice any physical activity during leisure time (0 min/week)^9^. This fact is worrisome, because sedentary lifestyle in adulthood is the fourth leading cause of death in the world^10^.

Additionally, adolescence is marked by intense transformations, both physiological and social. There may be more complex homework, parents divorced and greater demand of society regarding future plans. This complexity of factors can affect the adolescent's desire or ability to be physically active in daily life, as well as intensify their common mental disorder^1^.

To date, most studies have focused on adult populations in developed countries, sedentary behaviors (screen time) and patients with psychiatric illnesses. In addition, little is known about school physical education and sports practice in the context of common mental disorders^11^. In this sense, this study aimed to investigate the association of physical inactivity in leisure and school with common mental disorders in Brazilian adolescent students.

## METHODS

The Study of Cardiovascular Risks in Adolescents (Erica) is a cross-sectional, multicenter, national, school-based study that assessed adolescents aged between 12 and 17 years, enrolled in public and private schools (morning and afternoon classes), from Brazilian municipalities with more than 100,000 inhabitants. Data were collected from March 2013 to February 2014. Erica's protocol was previously described^12^.

The population under research was divided into 32 geographic strata comprising 27 capitals and five sets of municipalities with more than 100,000 inhabitants in each of the five geographical macro-regions of the country. Then a probabilistic sampling of schools was carried out in two stages. For each geographic stratum, we selected schools with probability proportional to size and inversely proportional to the distance from the capital. In the second stage, three classes in each school were selected with equiprobability during the field work, using the school year as an informative variable of age. The sample is representative for medium and large municipalities (> 100,000 inhabitants) at national and regional level and for all Brazilian capitals.

In the classes selected, all students who signed the consent term and brought the informed consent form signed by the guardians (when required by the local Research Ethics Committee) were interviewed and examined. Adolescents outside the elective age group, with physical or mental disabilities and pregnant were not considered eligible.

The data collection instrument was a self-completed questionnaire, applied in the classroom, under the supervision of the study team, through an individual and portable electronic data collector PDA (personal digital, assistant, model LG® GM750Q). To characterize the sample studied, the adolescents answered questions about gender (male or female), age in complete years (later categorized: 12–14 and 15–17 years), declared skin color, type of school (public or private), sexual maturation (self-assessment based on five stages)^13^ and weight status, from the body mass index curves for age proposed by the World Health Organization in 2007.

To determine the level of physical activity of adolescents, an adaptation of the self-administered physical activity checklist, was used, which consists of a list of 24 modalities and allows the adolescent to inform the frequency (days) and time (hours and minutes) they practiced, in the last week, any of the activities listed^14^. The version of this questionnaire used in Erica was validated in Brazilian adolescents^15^. In the estimation of the total time of physical activity, moderate to vigorous intensity modalities were considered (greater than or equal to three metabolic equivalents [MET]).

The product between time and frequency in each activity and the sum of the times obtained were estimated to determine the level of physical activity. Adolescents who accumulated at least 300 minutes/week of physical activity were considered active during leisure time, based on the level recommended by the WHO^16^. Individuals who did not report any leisure time physical activity in the week before the study (0 minute/week) were considered inactive. And those whose accumulated time in the previous seven days was higher than 0 and lower than 300 minutes were called insufficiently active^17^.

Participation in the physical education classes at school (yes/no and number of times per week) and the practice of sports outside/in the school (yes/no) were also assessed. Of the 24 modalities assessed to determine the adolescents’ level of physical activity, 12 were considered sports: athletics, swimming, martial arts (such as judo and karate), tennis, gymnastics, soccer, futsal, beach soccer, handball, basketball, court volleyball and beach volleyball.

Common mental disorders were assessed using the general health questionnaire (GHQ-12), a psychiatric screening instrument^5^. The GHQ-12 assesses mental health status and psychological distress without psychotic character, from 12 questions referring to the previous two weeks, with four answer options (more than usual, the same as usual, less than usual, much less than usual or much more than usual). The scores of the individual items were coded using a dichotomous scale. The answers with 1 or 2 points indicated absence (0), and the answers with 3 or 4 points, presence (1). Then all items were summed up. Using this method, the participant was able to reach from 0 to 12 points; scores of 3 or more^3^ indicated the presence of common mental disorders, which poses risk for the development of psychiatric disorders^4^. The GHQ-12 was validated for the Brazilian population, with a psychiatric interview structured as gold standard^18^.

Measures of relative frequency (prevalence) of a score equal to or greater than 3 were estimated for the different categories of leisure time of physical activity (active, insufficiently active and inactive), for sports practice (yes/no) and for the practice of physical education at school (yes/no and by number of times/week). Considering the sample design, odds ratios (OR), with their respective 95% confidence intervals (95%CI), were estimated by simple logistic regression adjusted by gender, age, type of school, stage of sexual maturation, body mass index, smoking (smoked at least once in the last thirty days) and alcohol consumption (consumed at least one glass or dose of alcoholic beverage in the last thirty days). The survey module of the Stata version 12.0 (StataCorp) program was used for complex sample data analysis, since it employs stratification and conglomeration in its selection stages.

Erica was carried out in accordance with the principles of the Declaration of Helsinki. The study was approved by the Research Ethics Committees of the Universidade Federal do Rio de Janeiro (Process 45/2008) and each federation unit.

## RESULTS

A total of 73,399 adolescents (71.7% of eligible students) from 1,247 schools in 124 Brazilian municipalities were evaluated. National coverage of eligible adolescents was 74.8% (n = 40,675) in females and 68.3% (n = 32,724) in males. Thirty-six-year-old students with physical disabilities that prevented the measures, and 215 pregnant women were not considered eligible, representing 0.6% of the total sample aged 12 to 17 years. The refusal to participate in the study was higher in male adolescents in all age groups. The participation of younger people was always higher than that of older people in both male and female genders.

Table 1 shows most of the population is composed of individuals with brown skin color (52.5%), from public schools (78.7%) and adequate weight status (72.6%). Average age was 14.7 years (standard deviation (SD) = 1.6). Less than half of the sample (44.4%) was composed of active adolescents (≥ 300 minutes/week), 29.1% reported no leisure time physical activity (0 minute/week), and 26.5% were considered insufficiently active (1–299 minutes/week). A significant proportion of the young people did not practice any sport (52.4%). Regarding physical education at school, 77.6% participated in classes, and 40.6% did once/week; 32.6%, twice/week; and 4.4%, three times/week.

**Table 1:** Characterization of the sample of Brazilian adolescents, according to sociodemographic variables, sexual maturation and weight status. Erica, Brazil, 2013–2014 (n = 73,399).

Variables		%
Gender		
	Boys	44.6
	Girls	55.4
Age (years)		
	12–14	45.9
	15–17	54.1
Skin color		
	Black	7.8
	Mixed race	52.5
	White	36.4
	Yellow	2.6
	Indigenous	0.7
Type of school		
	Public	78.7
	Private	21.3
Stage of sexual maturation^a^		
	Prepubescent	0.5
	Pubescent	62.9
	Postpubescent	36.6
Weight status^b^		
	Underweight	2.9
	Adequate	72.6
	Overweight	16.8
	Obesity	7.7
Common mental disorders		
	Yes	31.8
	No	68.2

a Prepubescent (Tanner stages 1 and 2), pubescent (Tanner stage 3) and postpubescent (Tanner stages 4 and 5).

b Classification of nutritional status according to z score of body mass index for age^33^.

Table 2 shows the association of common mental disorders with the volume of leisure time physical activity and sports practice. The frequency of individuals with a score ≥ 3 in the GHQ-12 was higher in the group of adolescents who did not practice sports (OR = 1.14) and did not perform any leisure time physical activity (0 minute/week) (OR = 1.16), when compared with active young people.

**Table 2:** Prevalence of Brazilian adolescents with common mental disorders/CMD (GHQ ≥ 3) and corresponding odds ratios (OR), according to leisure time physical activity and sports practice. Erica, Brazil, 2013–2014.

		Total (n)	With CMD (%)	Gross OR (95%CI)	Adjusted OR^b^ (95%CI)
Leisure time physical activity^c^					
	No	13,334	38.5	1.00 (reference)	1.00 (reference)
	Yes	60,065	30.4	0.64 (0.58–0.70)^a^	0.74 (0.66–0.82)^a^
Leisure time physical activity					1.00
	Active (≥ 300 minutes/week)	32,613	28.4	1.00 (reference)	(reference)
	Insufficiently active (1–299 minutes/week)	19.453	30.4	1.07 (0.95–1.19)	0.92 (0.82–1.03)
	Inactive (0 minute/week)	21,333	38.4	1.58 (1.46–1.69)^a^	1.16 (1.06–1.27)^a^
Sports practice^d^					
	Yes	34,932	25.9	1.00 (reference)	1.00 (reference)
	No	38,467	37.2	1.63 (1.52–1.74)^a^	1.14 (1.04–1.25)^a^

95%CI: 95% confidence interval.

a p < 0.01.

b Multiple logistic regression model, adjusted for gender, age, type of school, stage of sexual maturation, body mass index, smoking and alcohol consumption.

c Practice of any physical activity during leisure time, regardless of modality, duration or weekly frequency.

d Practice of one of the following modalities: athletics, swimming, tennis, wrestling, gymnastics, soccer/futsal, handball, basketball and volleyball.

Insufficient physical activity (1–299 minutes/week) did not increase the OR of common mental disorders. Likewise, no significant increase was found in OR after subdivision of insufficiently active adolescents into two categories (1–149 minutes/week and 150–299 minutes/week). When compared with individuals who did not practice any leisure time physical activity, those insufficiently active were less likely to have common mental disorders (adjusted OR = 0.79; 95%CI 0.70–0.89).

The practice of leisure time physical activity, regardless of modality, duration or weekly frequency, reduced by 26% the chances of common mental disorders in this population (Table 2), while the practice in accordance with the recommendation of at least 300 minutes/week decreased by 14% (adjusted OR = 0.86; 95%CI 0.79–0.94).

Regarding the practice of physical education at school (Table 3), an inverse relationship was observed between participation in classes and common mental disorders (p < 0.01). The prevalence of common mental disorders was higher among young people who did not participate in physical education classes (39.5%), compared with those who did (29.6%).

**Table 3:** Prevalence of Brazilian adolescents with common mental disorders/CMD (GHQ ≥ 3) and corresponding odds ratios (OR), according to participation in physical education classes at school. Erica, Brazil, 2013–2014.

		Total (n)	With CMD (%)	Gross OR (95%CI)	Adjusted OR^b^ (95%CI)
Participation in physical education classes^c^					
	Yes	56,920	29.6	1.00 (referência)	1.00 (referência)
	No	16,479	39.5	1.56 (1.45–1.68)^a^	1.25 (1.15–1.36)^a^
Frequency of physical education at school					
	Did not participate	16,479	39.5	1.00 (referência)	1.00 (referência)
	1 time per week	29,791	31.3	0.71 (0.65–0.77)^a^	0.85 (0.77–0.93)^a^
	2 times per week	23,929	27.8	0.58 (0.54–0.63)^a^	0.74 (0.68–0.82)^a^
	3 times per week	3,200	27.5	0.63 (0.53–0.73)^a^	0.91 (0.76–1.09)

95%CI: 95% confidence interval.

a p < 0.01.

b Multiple logistic regression model, adjusted for gender, age, type of school, stage of sexual maturation, body mass index, smoking and alcohol consumption.

c participation in physical education classes at school at least once per week.

## DISCUSSION

This is the first epidemiological study conducted in Brazil, with national representativeness, which assessed the relationship between common mental disorders and levels of physical activity during adolescence. The research produced three results for discussion. First, the frequency of common mental disorders was higher among adolescents who did not play sports, did not participate in physical education classes at school and were inactive (0 minute/week). According to the result, insufficient physical activity (1–299 minutes/week) did not increase the OR of common mental disorders in this population. Third, we observed that the practice of physical activity in accordance with the recommendation of at least 300 minutes/week reduced the OR for common mental disorders but did not offer additional benefit to the mental health of young people. A significant reduction in the chance of common mental disorders was observed with leisure time physical activity regardless of modality, duration and weekly frequency.

The few currently available evidence on the subject has shown the negative impact of insufficient physical activity on the mental health of young people^19^^,^^20^. A study conducted in Norway with adolescents aged 12 to 15 years showed a cross-sectional and longitudinal relation between low levels of vigorous activity and depressive symptoms (using the Mood and Feelings Questionnaire)^19^. Prospective cohort (2004–2010), conducted in Denmark, presented similar results, but only for females. Girls with low level of leisure time physical activity at 14/15 years had an increased risk of 60% for mental health problems at 20/21 years, compared with those with high level (> 4 hours/week)^20^.

The results of this study are in line with a systematic review of prospective studies involving children, adolescents and adults (1988–2012), which suggested any level of physical activity, such as walking less than 150 minutes/week, to prevent depression (defined by cutoff points on self-report scales or medical diagnosis)^21^. Recent research has also provided encouraging support for physical activity as beneficial intervention in mood. Thirty minutes of running at moderate intensity during the morning, for three consecutive weeks, positively affected concentration during the day and psychological functioning of adolescents, compared with control individuals^22^.

A similar beneficial effect was observed in a study with Australian adolescents, which showed a 9% decrease in the probability of depressive symptoms among boys who practiced moderate to vigorous activities daily for more than one hour^6^. Alghadir et al.^23^, studying individuals aged 7 to 18 years, concluded that the practice of physical activity is important for the improvement of depressive symptoms (Children's Depression Inventory scale),in addition to increasing serotonin levels and reducing serum cortisol.

This study showed a positive association between leisure time physical activity and mental health, regardless of modality, duration or frequency. Thus, public policies could consider the inclusion of the mental health dimension of totally inactive young people (0 minute/week), since there seems to be no additional benefit for mental health associated with compliance with the levels of activity recommended by the WHO. Scientific evidence show that the physical health of adolescents is improved by frequent physical activity^24^.

The findings of this study showed a higher occurrence of common mental disorders in the portion of the population that did not practice sports. Corroborating our study, Sabiston et al.^25^ found that participation in team sports during high school can protect against depressive symptoms in early adulthood, probably because team sport provides group work, opportunity for interaction with people and better perceived social acceptance. Leisure time physical activity and participation in sports also contributed to a greater sense of well-being and lower levels of anxiety and depression among European adolescents^26^.

Regarding physical activity at school, we observed that participation in physical education classes was negatively associated with common mental disorders in Erica's sample. A cross-sectional study with Australian adolescents (10–16 years) also detected that being more active in school, participating in physical education (OR = 0.77; 95%CI 0.69–0.86) and sports teams (OR = 0.77; 95%CI 0.67–0.88) may decrease the OR for depressive symptoms^27^. From this perspective, the school is a privileged space for adolescents to perform physical activities, considering that it is a safe place, with available equipment and appropriate professional guidance^9^. However, it is noteworthy the absence of a clear dose-response relation between the level of physical activity and the presence of common mental disorders.

The relation between mental well-being and physical activity has drawn the attention of researchers; however, some studies have not shown consistent associations. According to Rothon et al.^28^, each additional hour of physical activity undertaken during the week decreases the probability of depressive symptoms in adolescence by about 8%. However, the relation between these variables was not significant in a longitudinal analysis after two years of follow-up. A cohort study in the United Kingdom also did not support the hypothesis that physical activity, objectively measured by heart rate and motion detection, prevents the development of depressive symptoms (Mood and Feelings Questionnaire) during adolescence^29^.

Several hypotheses have been proposed to explain the protective effect of physical activity. Biological mechanisms, such as increased levels of monoamines in the circulation (norepinephrine, dopamine and serotonin) and lower cortisol secretion, may explain the antidepressant action of physical activity^23^. In addition, the fulfillment of goals and challenges of motor activity causes a feeling of self-efficacy, improves self-esteem and generates distraction from daily stressors^19^. The social network built on sports activity can also be important, just as sedentary behavior can reduce social interactions among individuals, causing loneliness^30^.

Otherwise, depressive symptoms, even below the threshold of clinical diagnosis, apathy and feelings of hopelessness can initiate a negative pattern in which it is difficult to gather energy and motivation to start exercising^19^. The protective effect of physical activity and the inhibiting effect of depressed mood may also coexist. Data from a longitudinal study with girls aged 11 to 15 years, assessed annually for six years, indicated a bidirectional relationship between physical activity and depression. Physical activity significantly reduced both the risk of major depressive disorder and the risk of future depressive symptoms, and vice versa^31^.

One of the limitations of this study was the use of a questionnaire to determine the adolescents’ level of physical activity. Although this instrument has been validated^15^, it does not always produce accurate estimates, and its agreement with direct measures is partial^32^. However, the use of direct measurement methods, such as accelerometry, would be unfeasible in a study such as Erica, due to its high cost and complexity. The variety of instruments and cut-off points (to measure common mental disorders and levels of physical activity) in the scientific literature also limited our study, making it difficult to compare with our results. THE GHQ-12 is a screening tool, and therefore it was not possible to establish a formal diagnosis of psychopathologies.

Another limitation is related to the cross-sectional nature (reverse causality). It is also noteworthy that the use of OR may overestimate the strength of the association in cross-sectional studies, when the outcome has a high prevalence in the population, which possibly occurred in this study. In addition, another question that discusses the plausibility of the association having a causal relationship is the relatively small magnitude of the association. The power of the sample could identify magnitudes even lower than those found in this study, which reflects on the relevance/property of making more incisive recommendations. On the other hand, the strengths of this study include the large and representative sample for Brazilian adolescents, the use of validated questionnaires, the high participation rate, and the analyses adjusted for several confounding factors (gender, age, type of school, stage of sexual maturation, body mass index, smoking and alcohol use).

In conclusion, common mental disorders are associated with physical inactivity at leisure (0 minute/week) and at school during adolescence. Leisure time physical activity, even without reaching the current health recommendation (≥ 300 minutes/week), may reduce the OR of common mental disorders, according to the data of this study. Sports practice and school physical education can also have beneficial effects on the mental health of young people. Erica's results reinforce the need to transition from total inactivity to regular physical activity, regardless of weekly frequency and volume of the physical activity initially practiced.
