# Supplementary material for: Physical inactivity during leisure and school time is associated with the presence of common mental disorders in adolescence
Source: Rev Saude Publica. 2020 Nov 23;54:128. doi: 10.11606/s1518-8787.2020054001888 (PMC7688259; doi:10.11606/s1518-8787.2020054001888)
Supplement: Translation

# Translation: Inatividade física no lazer e na escola está associada à presença de transtornos mentais comuns na adolescência

**Authors:** Vanessa Roriz Ferreira, Thiago Veiga Jardim, Thaís Inácio Rolim Póvoa, Ricardo Borges Viana, Ana Luiza Lima Sousa, Paulo César Veiga Jardim

**Affiliations:** I GoiâniaGO Brasil Universidade Federal de Goiás. Faculdade de Medicina. Liga de Hipertensão Arterial. Goiânia, GO, Brasil; II BostonMA USA Brigham & Women's Hospital. Division of Cardiovascular Medicine. Boston, MA, USA; III BostonMA USA Harvard TH Chan School of Public Health. Department of Health Policy and Management. Center for Health Decision Science, Boston, MA, USA; IV GoiâniaGO Brasil Universidade Estadual de Goiás. Laboratório de Fisiologia do Exercício da Faculdade do Esporte ESEFFEGO. Goiânia, GO, Brasil

**Keywords:** Adolescente, Comportamento Sedentário, Transtornos Mentais, Saúde Mental, Educação Física e Treinamento

## Abstract

**OBJETIVO:**

Investigar a associação da inatividade física no lazer e na escola com os transtornos mentais comuns durante a adolescência.

**MÉTODOS:**

A amostra foi composta por 73.399 adolescentes (12–17 anos), participantes do Estudo de Riscos Cardiovasculares em Adolescentes (Erica). Esse estudo transversal, nacional e de base escolar foi realizado em 2013 e 2014, nos municípios brasileiros com mais de 100 mil habitantes. A atividade física no lazer foi categorizada de acordo com o volume de prática semanal, e os adolescentes foram classificados em ativos (≥ 300 minutos/semana), inativos (0 minuto/semana) e insuficientemente ativos (1–299 minutos/semana). A prática esportiva e a participação em aulas de educação física na escola também foram analisadas. A presença de transtornos mentais comuns foi avaliada a partir do *general health questionnaire*, com ponto de corte maior ou igual a 3. *Odds ratios* (OR) foram estimados utilizando regressão logística múltipla.

**RESULTADOS:**

A chance de transtornos mentais comuns foi 16% maior no grupo que relatou ser inativo (0 minuto/semana) no lazer (OR = 1,16; IC95% 1,06–1,27). A prevalência de transtornos mentais comuns foi maior entre jovens que não praticaram esportes (37,2% *vs.* 25,9%; OR = 1,14; IC95% 1,04–1,25) e não participaram das aulas de educação física na escola (39,5% *vs.* 29,6%; OR = 1,25; IC95% 1,15–1,36). Atividade física insuficiente (1–299 minutos/semana) não aumentou a razão de chances de transtornos mentais comuns. Praticar atividade física no lazer, independentemente da duração e frequência semanal, reduziu em 26% as chances de transtornos mentais comuns nessa população.

**CONCLUSÕES:**

A inatividade física no lazer e na escola foi associada à presença de transtornos mentais comuns na adolescência. Os resultados sugerem que prática esportiva, educação física escolar e atividade física durante o lazer, mesmo sem atingir a recomendação atual, têm relação com a saúde mental dos jovens.

## INTRODUÇÃO

Dados epidemiológicos mostram que até 20% das crianças e adolescentes sofrem de uma doença mental incapacitante e que cerca de 50% dos transtornos mentais têm início na adolescência^1^. Mundialmente, os transtornos neuropsiquiátricos são a principal causa de anos de vida perdidos por incapacidade na faixa etária entre 10 e 24 anos^2^. No Brasil, dados do Estudo de Riscos Cardiovasculares em Adolescentes (Erica) relataram elevada prevalência (30,0%) de transtornos mentais comuns na população jovem^3^.

Os transtornos mentais comuns, também conhecidos como distúrbios psiquiátricos menores ou sofrimento psíquico, são desordens mentais sem caráter psicótico não contempladas nos critérios diagnósticos dos sistemas de classificação categórica. São caracterizados principalmente pela presença de sintomas de depressão e ansiedade, além de queixas somáticas inespecíficas. Esses transtornos, que podem ser manifestações iniciais de doenças psiquiátricas mais graves, prejudicam a vida escolar e as relações sociais do adolescente^4^^,^^5^.

Evidências recentes relacionaram sintomas de depressão e ansiedade com inatividade física^6^^,^^7^. Estudo transversal com adolescentes australianos mostrou que meninos com baixo nível de atividade física estão mais propensos à sintomatologia depressiva^6^. Por outro lado, pesquisa longitudinal conduzida na Alemanha revelou que mulheres jovens (12–26 anos), ao praticarem atividade física por mais tempo que o usual, sentiam-se menos deprimidas na manhã do dia seguinte^7^.

Ser ativo durante a adolescência também aumenta a chance de se manter ativo na idade adulta, previne doenças cardiovasculares, melhora a composição corporal e aumenta a força muscular, além dos benefícios imediatos ao jovem, tais como sensação de bem-estar e maior autoestima^8^. Apesar disso, no Brasil, um em cada quatro adolescentes não pratica nenhuma atividade física no lazer (0 min/semana)^9^. Este fato é preocupante, pois o estilo de vida sedentário na vida adulta é a quarta maior causa de mortes no mundo^10^.

Adicionalmente, a vida na adolescência é marcada por intensas transformações, tanto fisiológicas quanto sociais. É possível que ocorram trabalhos escolares de maior complexidade, separação dos pais e maior exigência da sociedade referente a planos futuros. Essa complexidade de fatores pode afetar o desejo ou a capacidade do adolescente de ser fisicamente ativo na vida diária, bem como intensificar seu transtorno mental comum^1^.

Até a data, a maioria das pesquisas tem focado em populações adultas de países desenvolvidos, nos comportamentos sedentários (tempo em atividades de tela) e nos portadores de enfermidades psiquiátricas. Além disso, pouco se sabe sobre a educação física escolar e a prática esportiva no contexto dos transtornos mentais comuns^11^. Nesse sentido, o presente estudo teve como objetivo investigar a associação de inatividade física no lazer e na escola com transtornos mentais comuns em adolescentes escolares brasileiros.

## MÉTODOS

O Estudo de Riscos Cardiovasculares em Adolescentes (Erica) é um estudo transversal, multicêntrico, nacional, de base escolar, que avaliou adolescentes com idades entre 12 e 17 anos, matriculados em escolas públicas e privadas (período matutino e vespertino), de municípios brasileiros com mais de 100.000 habitantes. Os dados foram coletados entre março de 2013 e dezembro de 2014. O protocolo do Erica foi previamente descrito^12^.

A população da pesquisa foi dividida em 32 estratos geográficos, constituídos por 27 capitais e cinco conjuntos de municípios com mais de 100 mil habitantes em cada uma das cinco macrorregiões geográficas do país. Em seguida, realizou-se uma amostragem probabilística de escolas em duas etapas. Na primeira, foram selecionadas escolas para cada estrato geográfico, com probabilidade proporcional ao seu tamanho e inversamente proporcional à distância da capital. Na segunda etapa, três turmas em cada escola sorteada foram selecionadas com equiprobabilidade durante o trabalho de campo, usando o ano da turma como variável informativa da idade. A amostra é representativa para municípios de médio e grande porte (> 100 mil habitantes) em âmbito nacional e regional e para as capitais brasileiras.

Nas turmas selecionadas, foram entrevistados e examinados todos os alunos que assinaram o termo de assentimento e trouxeram o termo de consentimento livre e esclarecido assinado pelos responsáveis (quando exigido pelo Comitê de Ética em Pesquisa local). Foram excluídos das análises, por não serem considerados elegíveis, adolescentes fora da faixa etária eletiva, com deficiência física ou mental e grávidas.

O instrumento de coleta dos dados foi um questionário autopreenchível, aplicado em sala de aula, sob supervisão da equipe do estudo, por meio de um coletor eletrônico de dados individual e portátil PDA (*personal digital assistant*, modelo LG^®^ GM750Q). Para caracterização da amostra estudada, os adolescentes responderam a questões sobre sexo (masculino ou feminino), idade em anos completos (posteriormente categorizada: 12–14 e 15–17 anos), cor da pele declarada, tipo de escola (pública ou privada), maturação sexual (autoavaliação baseada em cinco estágios)^13^ e estado de peso, a partir das curvas de índice de massa corporal para idade propostas pela Organização Mundial de Saúde em 2007.

Para determinação do nível de atividade física dos adolescentes, foi utilizada uma adaptação do *self-administered physical activity checklist*, que consiste em uma lista de 24 modalidades e permite que o adolescente informe a frequência (dias) e o tempo (horas e minutos) que praticou, na última semana, alguma das atividades listadas^14^. A versão desse questionário utilizada no Erica foi validada em adolescentes brasileiros^15^. No cálculo do tempo total de atividade física, consideraram-se modalidades de intensidade moderada a vigorosa (maior ou igual a três equivalentes metabólicos [METs]).

O produto entre o tempo e a frequência em cada atividade e o somatório dos tempos obtidos foram calculados para determinar o nível de atividade física. Os adolescentes que acumularam pelo menos 300 minutos/semana de atividade física foram considerados ativos no lazer, com base no nível recomendado pela OMS^16^. Os indivíduos que não referiram qualquer prática de atividade física no lazer na semana anterior à pesquisa (0 minuto/semana) foram considerados inativos. E aqueles cujo tempo acumulado nos últimos sete dias foi maior que 0 e menor que 300 minutos foram denominados insuficientemente ativos^17^.

A participação nas aulas de educação física da escola (sim/não e número de tempos por semana) e a prática esportiva fora/dentro da escola (sim/não) também foram avaliadas. Das 24 modalidades avaliadas para determinação do nível de atividade física dos adolescentes, 12 foram consideradas modalidades esportivas: atletismo, natação, lutas (como judô e caratê), tênis, ginástica olímpica, futebol, futsal, futebol de praia, handebol, basquete, vôlei de quadra e vôlei de praia.

Os transtornos mentais comuns foram avaliados por meio do *general health questionnaire* (GHQ-12), um instrumento de rastreamento psiquiátrico^5^. O GHQ-12 avalia o estado de saúde mental e o sofrimento psíquico sem caráter psicótico, a partir de 12 questões referentes às duas semanas precedentes, com quatro opções de resposta (mais que de costume, o mesmo de sempre, menos que de costume, muito menos que de costume ou muito mais que de costume). Os escores dos itens individuais foram codificados por meio de uma escala dicotômica. As respostas com 1 ou 2 pontos indicaram ausência (0), e as respostas com 3 ou 4 pontos, presença (1). Em seguida, todos os itens foram somados. Usando esse método, o participante pôde alcançar de 0 a 12 pontos; escores de 3 ou mais^3^ indicaram a presença de transtornos mentais comuns, que refere-se a um estado de risco para o desenvolvimento de distúrbios psiquiátricos^4^. O GHQ-12 foi validado para a população brasileira, tendo uma entrevista psiquiátrica estruturada como padrão ouro^18^.

Foram calculadas medidas de frequência relativa (prevalências) de escore igual ou maior que 3 para as diferentes categorias de tempo de atividade física no lazer (adolescentes ativos, insuficientemente ativos e inativos), para a prática esportiva (sim/não) e para a prática de educação física na escola (sim/não e por número de tempos/semana). Considerando o desenho amostral, *odds ratios* (OR), com seus respectivos intervalos de 95% de confiança (IC95%), foram estimados por meio de regressão logística simples e ajustada por sexo, idade, tipo de escola, estágio de maturação sexual, índice de massa corporal, tabagismo (fumou pelo menos uma vez nos últimos trinta dias) e consumo de álcool (consumiu pelo menos um copo ou dose de bebida alcoólica nos últimos trinta dias). Utilizou-se o módulo *survey* do programa Stata versão 12.0 (StataCorp) para análise de dados de amostra complexa, uma vez que emprega estratificação e conglomeração em seus estágios de seleção.

O Erica foi realizado de acordo com os princípios da Declaração de Helsinque. O estudo foi aprovado pelos Comitês de Ética em Pesquisa da Universidade Federal do Rio de Janeiro (Processo 45/2008) e de cada unidade da federação.

## RESULTADOS

Foram avaliados 73.399 adolescentes (71,7% dos alunos elegíveis), de 1.247 escolas, em 124 municípios brasileiros. A cobertura nacional dos adolescentes elegíveis foi de 74,8% (n = 40.675) no sexo feminino e 68,3% (n = 32.724) no sexo masculino. Não foram considerados elegíveis 364 escolares com deficiência física que inviabilizava as medidas e 215 grávidas, representando 0,6% do total da amostra de 12 a 17 anos. A recusa de participação no estudo foi maior nos adolescentes do sexo masculino em todas as faixas etárias. A participação dos mais jovens foi sempre maior do que a dos mais velhos, em ambos os sexos.

A Tabela 1 mostra que a maioria da população é composta por indivíduos com cor da pele parda (52,5%), de escolas públicas (78,7%) e estado de peso adequado (72,6%). A média de idade foi de 14,7 (desvio padrão = 1,6) anos. Menos da metade da amostra (44,4%) foi composta por adolescentes ativos (≥ 300 minutos/semana), 29,1% reportaram nenhuma atividade física no lazer (0 minuto/semana), e 26,5% foram considerados insuficientemente ativos (1–299 minutos/semana). Uma parcela significativa dos jovens não praticava nenhum esporte (52,4%). Sobre a educação física na escola, 77,6% participavam das aulas, sendo que 40,6% faziam um tempo/semana; 32,6%, dois tempos/semana; e 4,4%, três tempos/semana.

**Tabela 1:** Caracterização da amostra de adolescentes brasileiros, segundo variáveis sociodemográficas, maturação sexual e estado de peso. Erica, Brasil, 2013–2014 (n = 73.399).

Variáveis		%
Sexo		
	Meninos	44,6
	Meninas	55,4
Idade (anos)		
	12–14	45,9
	15–17	54,1
Cor da pele		
	Preta	7,8
	Parda	52,5
	Branca	36,4
	Amarela	2,6
	Indígena	0,7
Tipo de escola		
	Pública	78,7
	Privada	21,3
Estágio de maturação sexual^a^		
	Pré-púberes	0,5
	Púberes	62,9
	Pós-púberes	36,6
Estado de peso^b^		
	Baixo peso	2,9
	Adequado	72,6
	Sobrepeso	16,8
	Obesidade	7,7
Transtornos mentais comuns		
	Sim	31,8
	Não	68,2

a Pré-púberes (estágios de Tanner 1 e 2), púberes (estágio de Tanner 3) e pós-púberes (estágios de Tanner 4 e 5).

b Classificação do estado nutricional, segundo escore z de índice de massa corporal para a idade^33^.

Na Tabela 2 pode-se observar a associação dos transtornos mentais comuns com o volume de atividade física no lazer e com a prática esportiva. A frequência de indivíduos com pontuação ≥ 3 no GHQ-12 foi maior no grupo dos adolescentes que não praticaram esportes (OR = 1,14) e não realizaram nenhuma atividade física no lazer (0 minuto/semana) (OR = 1,16), quando comparados aos jovens ativos.

**Tabela 2:** Prevalência de adolescentes brasileiros com transtornos mentais comuns/TMC (GHQ ≥ 3) e *odds ratios* (OR) correspondentes, de acordo com a atividade física no lazer e a prática esportiva. Erica, Brasil, 2013–2014.

		Total (n)	Com TMC (%)	OR bruto (IC95%)	OR ajustado^b^ (IC95%)
Prática de atividade física no lazer^c^					
	Não	13.334	38,5	1,00 (referência)	1,00 (referência)
	Sim	60.065	30,4	0,64 (0,58–0,70)^a^	0,74 (0,66–0,82)^a^
Tempo de atividade física no lazer					
	Ativos (≥ 300 minutos/semana)	32.613	28,4	1,00 (referência)	1,00 (referência)
	Insuficientemente ativos (1–299 minutost/semana)	19,453	30,4	1,07 (0,95–1,19)	0,92 (0,82–1,03)
	Inativos (0 minuto/semana)	21.333	38,4	1,58 (1,46–1,69)^a^	1,16 (1,06–1,27)^a^
Prática esportiva^d^					
	Sim	34.932	25,9	1,00 (referência)	1,00 (referência)
	Não	38.467	37,2	1,63 (1,52–1,74)^a^	1,14 (1,04–1,25)^a^

IC95% = intervalo de confiança de 95%.

a p < 0,01.

b Modelo de regressão logística múltipla, ajustado por sexo, idade, tipo de escola, estágio de maturação sexual, índice de massa corporal, tabagismo e consumo de álcool.

c Prática de alguma atividade física no lazer, independentemente da modalidade, duração ou frequência semanal.

d Prática de uma das seguintes modalidades: atletismo, natação, tênis, lutas, ginástica olímpica, futebol/futsal, handebol, basquete e vôlei.

Atividade física insuficiente (1–299 minutos/semana) não aumentou a razão de chances de transtornos mentais comuns (Tabela 2). Do mesmo modo, não houve aumento significativo no OR após subdivisão dos adolescentes insuficientemente ativos em duas categorias (1–149 minutos/semana e 150–299 minutos/semana). Quando comparados com indivíduos que não praticaram nenhuma atividade física no lazer, os insuficientemente ativos tiveram menores chances de transtornos mentais comuns (OR ajustado = 0,79; IC95% 0,70–0,89).

A prática de atividade física durante o lazer, independentemente de modalidade, duração ou frequência semanal, reduziu em 26% as chances de transtornos mentais comuns nessa população (Tabela 2), enquanto a prática em conformidade com a recomendação de pelo menos 300 minutos/semana reduziu em 14% (ajustado = 0,86; IC95% 0,79–0,94).

Com relação à prática de educação física na escola (Tabela 3), foi observada relação inversa entre participação nas aulas e os transtornos mentais comuns (p < 0,01). A prevalência de transtornos mentais comuns foi maior entre jovens que não participavam das aulas de educação física escolar (39,5%), comparados com os que participavam da educação física escolar (29,6%).

**Tabela 3:** Prevalência de adolescentes brasileiros com transtornos mentais comuns/TMC (GHQ ≥ 3) e *odds ratios* (OR) correspondentes, de acordo com a participação em aulas de educação física na escola. Erica, Brasil, 2013–2014.

		Total (n)	Com TMC (%)	OR bruto (IC95%)	OR ajustado^b^ (IC95%)
Participação nas aulas de educação física^c^					
	Sim	56.920	29,6	1,00 (referência)	1,00 (referência)
	Não	16.479	39,5	1,56 (1,45–1,68)^a^	1,25 (1,15–1,36)^a^
Frequência da educação física na escola					
	Não participaram	16.479	39,5	1,00 (referência)	1,00 (referência)
	1 tempo por semana	29.791	31,3	0,71 (0,65–0,77)^a^	0,85 (0,77–0,93)^a^
	2 tempos por semana	23.929	27,8	0,58 (0,54–0,63)^a^	0,74 (0,68–0,82)^a^
	3 tempos por semana	3.200	27,5	0,63 (0,53–0,73)^a^	0,91 (0,76–1,09)

IC95%= intervalo de confiança de 95%.

a p < 0,01.

b Modelo de regressão logística múltipla, ajustado por sexo, idade, tipo de escola, estágio de maturação sexual, índice de massa corporal, tabagismo e consumo de álcool.

c Participação nas aulas de educação física da escola pelo menos um tempo por semana.

## DISCUSSÃO

Este é o primeiro estudo epidemiológico conduzido no Brasil, com representatividade nacional, que avaliou a relação entre transtornos mentais comuns e níveis de atividade física durante a adolescência. A pesquisa produziu três resultados para discussão. Primeiro, a frequência de transtornos mentais comuns foi maior entre adolescentes que não praticavam esportes, não participavam das aulas de educação física na escola e eram inativos (0 minuto/semana) no lazer. Segundo resultado, atividade física insuficiente (1–299 minutos/semana) não aumentou a razão de chances de transtornos mentais comuns nessa população. Terceiro, foi observado que a prática de atividade física em conformidade com a recomendação de pelo menos 300 minutos/semana reduziu a razão de chances para os transtornos mentais comuns, contudo não ofereceu benefício adicional para a saúde mental dos jovens. Redução significante na chance de transtornos mentais comuns foi observada com atividade física no lazer independentemente de modalidade, duração e frequência semanal.

As poucas evidências atualmente disponíveis sobre o assunto têm mostrado o impacto negativo de atividade física insuficiente na saúde mental da população jovem^19^^,^^20^. Estudo realizado na Noruega, com adolescentes de 12 a 15 anos, mostrou relação transversal e longitudinal entre baixos níveis de atividade vigorosa e sintomas depressivos (usando o *Mood and Feelings Questionnaire*)^19^. Coorte prospectiva (2004–2010), conduzida na Dinamarca, apresentou resultados semelhantes, mas somente para o sexo feminino. Meninas com baixo nível de atividade física no lazer aos 14/15 anos tiveram risco aumentado em 60% para problemas de saúde mental aos 20/21 anos, comparadas com as com alto nível (> 4 horas/semana)^20^.

Os resultados da presente pesquisa condizem com a revisão sistemática de estudos prospectivos envolvendo crianças, adolescentes e adultos (1988–2012), que sugeriram qualquer nível de atividade física, como caminhar menos de 150 minutos/semana, para prevenir depressão (definida por pontos de corte em escalas de autorrelato ou diagnóstico médico)^21^. Pesquisa recente também forneceu apoio encorajador para a atividade física enquanto intervenção benéfica no humor. Trinta minutos de corrida em intensidade moderada, durante a manhã, por três semanas consecutivas, impactaram positivamente na concentração durante o dia e no funcionamento psicológico de adolescentes, em comparação com indivíduos controle^22^.

Efeito benéfico similar foi observado em estudo com adolescentes australianos, que evidenciou um decréscimo de 9% na probabilidade de sintomas depressivos entre os meninos que praticavam atividades moderadas a vigorosas, diariamente, por mais de uma hora^6^. Alghadir e colaboradores^23^, estudando indivíduos de 7 a 18 anos, concluíram que a prática de atividade física é importante para a melhora dos sintomas depressivos (escala *Children's Depression Inventory*), além de aumentar os níveis de serotonina e reduzir o cortisol sérico.

Esse estudo apresentou associação positiva entre atividade física no lazer e saúde mental, independentemente de modalidade, duração ou frequência da prática. Assim, as políticas públicas poderiam considerar a inclusão da dimensão saúde mental dos jovens totalmente inativos (0 minuto/semana), já que parece não existir benefício adicional para a saúde mental associado ao cumprimento dos níveis de atividade recomendados pela OMS. Cabe ressaltar que há evidências científicas de que a saúde física dos adolescentes é melhorada por atividade física frequente^24^.

Os achados do presente estudo evidenciaram maior ocorrência de transtornos mentais comuns na parcela da população que não praticou esportes. Corroborando nosso estudo, Sabiston e colaboradores^25^ constataram que a participação em esportes de equipe durante o ensino médio pode proteger contra os sintomas depressivos no início da vida adulta, provavelmente pelo fato de o esporte em equipe proporcionar trabalho em grupo, oportunidade de interação com pessoas e melhor aceitação social percebida. Atividade física no lazer e participação em esportes também contribuíram para uma maior sensação de bem-estar e menores níveis de ansiedade e depressão entre adolescentes europeus^26^.

Com relação a atividade física na escola, foi observado que a participação em aulas de educação física foi negativamente associada a transtornos mentais comuns na amostra do Erica. Estudo transversal com adolescentes australianos (10–16 anos) detectou, do mesmo modo, que ser mais ativo na escola, participando da educação física (OR = 0,77; IC95% 0,69–0,86) e das equipes de esportes (OR = 0,77; IC95% 0,67–0,88), pode diminuir a razão de chances para os sintomas depressivos^27^. Nessa perspectiva, a escola é um espaço privilegiado para adolescentes realizarem atividades físicas, considerando que é um local seguro, com equipamentos disponíveis e orientação profissional apropriada^9^. Contudo, é importante destacar a ausência de uma relação dose-resposta clara entre o nível de atividade física e a presença de transtornos mentais comuns.

A relação entre bem-estar mental e atividade física tem atraído atenção de pesquisadores, entretanto alguns estudos não apresentaram associações consistentes. De acordo com Rothon e colaboradores^28^, cada hora adicional de atividade física empreendida na semana diminui em cerca de 8% a probabilidade de sintomas depressivos na adolescência. Todavia, a relação entre essas variáveis não foi significante em uma análise longitudinal, após dois anos de acompanhamento. Estudo de coorte no Reino Unido também não sustentou a hipótese de que atividade física, objetivamente mensurada por frequência cardíaca e detecção de movimento, evita o desenvolvimento de sintomas depressivos (*Mood and Feelings Questionnaire*) durante a adolescência^29^.

Várias hipóteses têm sido propostas para explicar o efeito protetor da atividade física. Mecanismos biológicos, como o aumento dos níveis de monoaminas na circulação (norepinefrina, dopamina e serotonina) e menor secreção de cortisol, podem explicar a ação antidepressiva da atividade física^23^. Além disso, o cumprimento de metas e desafios da atividade motora provoca o sentimento de autoeficácia, melhora a autoestima e gera distração dos estressores diários^19^. A rede social construída na atividade esportiva também pode ser importante, assim como o comportamento sedentário pode reduzir as interações sociais entre os indivíduos, gerando sentimentos de solidão^30^.

De outro modo, sintomas depressivos, mesmo abaixo do limiar de diagnóstico clínico, apatia e sentimentos de desesperança podem iniciar um padrão negativo em que é difícil reunir energia e motivação para iniciar a prática de atividade física^19^. O efeito protetor da atividade física e o efeito inibitório do humor deprimido também podem coexistir. Dados de um estudo longitudinal com meninas de 11 a 15 anos, avaliadas anualmente por um período de seis anos, sinalizaram uma relação bidirecional entre atividade física e depressão. A prática de atividade física reduziu significantemente tanto o risco de transtorno depressivo maior como o risco de sintomas depressivos futuros, e vice-versa^31^.

Uma das limitações deste estudo foi o uso de um questionário para determinar o nível de atividade física dos adolescentes. Apesar desse instrumento ter sido validado^15^, nem sempre produz estimativas precisas, e sua concordância com medidas diretas é parcial^32^. Contudo, a utilização de métodos de mensuração direta, como acelerometria, seria inviável em um estudo como o Erica, devido ao alto custo e à complexidade da logística envolvida. A variedade de instrumentos e pontos de corte (para mensurar os transtornos mentais comuns e os níveis de atividade física) na literatura científica também limitou o presente estudo, dificultando a comparação com nossos resultados. O GHQ-12 é uma ferramenta de triagem, e, por isso, não foi possível estabelecer diagnóstico formal de psicopatologias.

Outra limitação está relacionada à natureza transversal (causalidade reversa). Vale ressaltar, ainda, que o uso do OR pode superestimar a força da associação em estudos de delineamento transversal, quando o desfecho possui elevada prevalência na população, o que possivelmente ocorreu no presente estudo. Além disso, outra questão que coloca em discussão a plausibilidade da associação possuir relação causal é a relativamente pequena magnitude da associação. O poder da amostra poderia identificar magnitudes inclusive menores que as encontradas no presente estudo, o que faz refletir sobre a relevância/propriedade de se fazer recomendações mais incisivas. Por outro lado, os pontos fortes deste trabalho incluem a amostra grande e representativa para os adolescentes brasileiros, a utilização de questionários validados, a alta taxa de participação e as análises ajustadas por diversos fatores de confusão (sexo, idade, tipo de escola, estágio de maturação sexual, índice de massa corporal, tabagismo e uso de álcool).

Em conclusão, transtornos mentais comuns estão associados a inatividade física no lazer (0 minuto/semana) e na escola durante a adolescência. Atividade física no tempo de lazer, mesmo sem atingir a recomendação atual para a saúde (≥ 300 minutos/semana), pode reduzir a razão de chances de transtornos mentais comuns, de acordo com os dados do presente estudo. Prática esportiva e educação física escolar também podem ter efeitos benéficos na saúde mental dos jovens. Os resultados do Erica reforçam a necessidade de realizar a transição da inatividade total para uma prática regular de atividade física, independentemente da frequência semanal e do volume de atividade física inicialmente praticada.
